# Abstract Graphic Creativity, Feelings about School, and Engagement in the School Environment: What Are the Interindividual Differences between Gifted and Non-Gifted Children?

**DOI:** 10.3390/jintelligence11010002

**Published:** 2022-12-23

**Authors:** Christine Sanchez, Nathalie Blanc

**Affiliations:** Department of Psychology, Epsylon EA 4556, University Paul Valéry Montpellier 3, F34000 Montpellier, France

**Keywords:** giftedness, gifted children, school, autodetermination, creativity, feelings about school, schoolchildren

## Abstract

This study examines interindividual differences between gifted and non-gifted children in the school environment. Three distinct measurement tools were used to enable a multimodal approach of gifted and non-gifted children with abstract graphic creativity, feelings about school and engagement in the school environment being considered. The results obtained from 328 children (including 45 gifted children) revealed that the gifted children obtained scores significantly higher than non-gifted children in terms of self-determination, feelings about school, and creativity. In addition, a gap appears among gifted children between their significantly higher scores for motivation and intellectual skills versus standard scores for their socio-affective development. Such results are consistent with the idea of asynchronous development, which is characteristic of gifted children (cognitive and conative vs. socio-affective sphere), offering perspectives for more adapted support for these pupils in elementary school.

## 1. Introduction

This multimodal study addresses the general objective of understanding differences in functioning and perception between gifted and non-gifted children in a school setting. Based on the theoretical framework of intelligence developed by Renzulli (1978, 1988, 2002, 2016), the presence of interindividual differences between two populations (gifted vs. non-gifted children) deserves to be examined on three distinct components of school development. The first component concerns children’s commitment at school (academically and in terms of interpersonal relations), perceived and assessed by the teacher. The second component concerns children’s school experience through their feelings about school, including their relationship with their teacher. The third component has to do with children’s creativity, through the measurement of abstract graphic creativity. Understanding the differentiated functioning of the gifted child is indeed of practical interest in order to improve his support at school.

### 1.1. A Reminder about the Gifted Child

There is a lack of consensus concerning giftedness, which is reflected by the coexistence of several conceptions of the phenomenon as well as by the multiplicity of terminologies used to refer to it (e.g., Caroff 2004; Liratni and Pry 2007; Doi et al. 2019; Quartier et al. 2019). This difficulty in qualifying and defining giftedness is probably attributable to social taboos relating to the question of intelligence, since educational doctrine tends to favor an egalitarian approach (e.g., Mandelman et al. 2010). However, the diversity of approaches and profiles encountered (e.g., Francis et al. 2016) should not prevent the determination of giftedness constants, because the available scientific literature makes it possible to empirically distinguish several developmental characteristics shared by gifted children.

The gifted child, first of all, demonstrates exceptional development (i.e., particularly efficient and early) of his intellectual abilities (e.g., Lubart 2006; Papadopoulos 2020; Terrassier 2009; Winner et al. 1997) as they are evaluated at school or measured through intelligence tests by establishing an Intellectual Quotient (IQ). The most preferred intelligence test to date in France is the WISC-V for children aged 6 and up (WISC-V, Wechsler 2016; Caroff 2004; Delaubier 2002; Vrignaud 2002). Admittedly, these measures of academic and cognitive abilities are imperfect, because they only partially reflect human intelligence (Besançon and Lubart 2012) and prove problematic considering giftedness in particular. In this population, there would indeed be greater intraindividual and interindividual variability in the measurement of IQ, with a heterogeneity that is more characteristic than exceptional (Grégoire 2009; Liratni and Pry 2012; Pereira-Fradin et al. 2010; Quartier et al. 2019). The fact remains that in research, the calculation of a TIQ (i.e., the total IQ) serves as an objectification of the sampling of gifted children, using a threshold value which is normally 130 (e.g., Liratni and Pry 2007, 2011, 2012; Pereira-Fradin 2004; Pereira-Fradin et al. 2010; Pfeiffer and Jarosewich 2007), 125 (e.g., Aubry 2018; McIntosh et al. 2005; Norman et al. 1999; Simoes Loureiro et al. 2009), or elsewhere 120 (e.g., Katusic et al. 2011; Rost and Czeschlik 1994; Wellisch et al. 2011). Because intelligence is a continuum, perhaps the most important thing is that researchers select a cutoff value that is consistent with their theoretical approach to giftedness. In this study based on Renzulli’s theory of giftedness, which is particularly relevant in the educational setting (Da Costa 2019), it was important to choose an approach that was both inclusive and consistent with the Renzulli’s conception (Renzulli 1978, 1988, 2002, 2016). Cutoff values of 125 for an ITQ and 122 for a shortened version of the ITQ were thus selected.

Although it is now established from a neuroscientific point of view that gifted children (i.e., detected with intelligence tests) have better white matter integrity (Nusbaum et al. 2017), it is the singularity of their developmental construction which must serve in practice as a reference point for the detection of this population (Papadopoulos 2020). Indeed, some early developmental signs of gifted children could be rigorously identified, although a posteriori. In particular, these children would present an advanced rate of development before the age of one year, both in terms of motor and verbal acquisitions (Vaivre-Douret 2011, 2019). In addition, the observation of the gifted child’s sleep, which seems to be different (Bastien et al. 2021; Bergès-Bounes and Calmettes-Jean 2006; Revol and Bléandonu 2012), can also serve as an indicator. Furthermore, gifted children will often demonstrate a curiosity trait, with early and strong interests centered on existential questions (e.g., Lubart 2006; Romand and Weismann-Arcache 2018; Terrassier 2009; Winner et al. 1997).

Other behavioral traits remain under debate, such as anxiety (Tordjman and Kermarrec 2019), perfectionism (Stricker et al. 2020), and socio-emotional, affective, or communicative skills (Coleman and Cross 2005). It seems, however, that gifted children do not present superior socio-emotional faculties or deficits compared to their chronologically age-matched peers (e.g., Coleman and Cross 2005; Cross and Cross 2015; Francis et al. 2016; Lopez and Sotillo 2009; Papadopoulos 2020).

Two recent meta-analyses conducted on the emotional development of gifted children point to a significant but small difference in the emotional intelligence of these children (Abdulla Alabbasi et al. 2021; Ogurlu 2021; see also Schwean et al. 2006). These differences indicate a slightly better adaptability and mood of gifted children (Abdulla Alabbasi et al. 2021) or a better openness to experience (Ogurlu 2021). However, these differences remain small and highly dependent on the tools used to assess EI (e.g., Abdulla Alabbasi et al. 2021; Ogurlu 2021; Zeidner et al. 2005; Zeidner and Matthews 2017). The fact remains, therefore, that the development of gifted children often presents a characteristic gap between remarkably effective cognitive development and socio-emotional development close to that of their peers (e.g., Terrassier 2009). Such a discrepancy sometimes causes difficulties for these children, who reach an understanding of the complexity of the world before others, without being emotionally better equipped to confront it (e.g., Papadopoulos 2020). Because the different domains of human development (physical, cognitive, social, and emotional) are interdependent (Diamond 2007) and because emotional intelligence impacts both the emotional and social development of the child (Abdulla Alabbasi et al. 2021), emotional intelligence will be also discussed in this study, but mainly indirectly: The social cognition of the gifted schoolchild is indeed addressed through his perceived behavioral engagement with the teacher (AAS; Sanchez and Blanc 2019) and his perception of the fluidity of his relationship with the teacher (French FAS; Sanchez et al. 2022).

### 1.2. An Expanded Theoretical Framework

**Figure 1:**
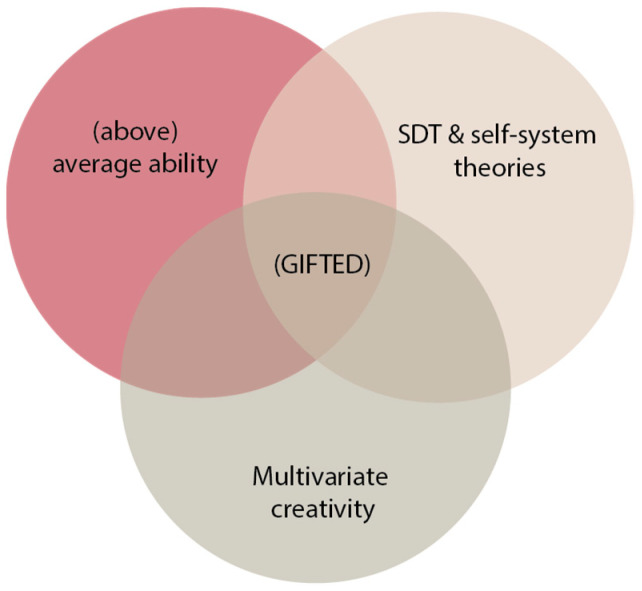
Graphical representation of intelligence in three rings, linked to theories of engagement and the multivariate approach to creativity.

Understanding the functioning of the gifted child means starting by stopping, in order to examine intelligence and the models that have been established to try to circumscribe this notion. We have therefore chosen to draw on several theoretical frameworks at the same time (i.e., the theories of commitment and the multivariate approach to creativity; Connell and Wellborn 1991; Ryan and Deci 2000, 2017; Sternberg and Lubart 1995, 1996) for an exhaustive look at the different components at play in the approach developed by Renzulli (e.g., Renzulli 1978, 1988, 2002, 2016). Particularly relevant to the educational context (Da Costa 2019), thanks to its interactional and dynamic character, Joseph Renzulli’s model of intelligence in three rings is the one that we have used as an anchor point (e.g., Renzulli 1978, 1988, 2002, 2016). According to this model, harmonious and performative development is located within the meeting zone of three key elements (see Figure 1): high intellectual abilities, creativity, and commitment. First, the three-ring theory is relevant for the optimization of both the potential and quality of life of the gifted child, at school as well as outside the school environment. Secondly, this model of intelligence is applicable to the harmonious development of the abilities of any child, regardless of his intellectual abilities at the outset. It is therefore on the basis of this theory that we have decided to compare the functioning of French gifted and non-gifted schoolchildren. Thus, our attention was focused equally on their level of academic success, commitment, and creativity.

Essential to the development of any child, commitment is defined by Joseph Renzulli (1978, 1988) as corresponding to the level of energy invested in a task (i.e., interest, enthusiasm, involvement, perseverance) or in a specific field of achievement. Taking into consideration Renzulli’s theory (e.g., Renzulli 1978, 1988, 2002, 2016), our intention was to consider each of these components in isolation, which is why we paid particular attention to the theories of engagement. The empirical macro-theory of SDT (Self-Determination Theory; Niemiec and Ryan 2009; Deci and Ryan 2000; Deci et al. 2017) emphasizes commitment as a prerequisite for the development of a productive appetite for learning (e.g., Joule 2004; Niemiec and Ryan 2009) and as a fundamental element of personality development (Ryan and Deci 2017). In this theory, the desire to learn seems innate and intrinsic, according to the Aristotelian idea that “man by nature wants to know”; however, there is a risk of seeing this appetite for learning dwindle when the conditions for its development are not met. Therefore, it is ideally necessary to encourage and/or awaken in the child his natural propensities to engage in learning so that this commitment persists in the school environment. In this sense, the theory of a self-system process or self-system theory (Connell and Wellborn 1991; Skinner and Belmont 1993; Skinner et al. 2008), derived from SDT (Ryan and Deci 2000, 2017) and particularly adapted to learning, maintains the importance of ensuring that the three fundamental psychological needs of the child learner are met: the need for a sense of academic competence, the need for autonomy, and the need for relationships (e.g., Valeski and Stipek 2001; Skinner et al. 2013; Tian et al. 2018; Sanchez et al. 2022). Two scales thus make it possible to better evaluate the pupil’s commitment. The first is a self-evaluating scale for measuring the schoolchild’s Feelings about School, the French version of the FAS called SSE (*Sentiments Sur l’Ecole* or French FAS; Sanchez et al. 2022), which is an enriched version of the original FAS scale (Valeski and Stipek 2001). The second is a heteroevaluative scale where we enlist the help of the teacher to measure the AAS (Autodetermination At School; Sanchez and Blanc 2019) of the schoolchild and to understand, in detail, their academic commitment and intrinsic motivation, as well as their behavioral involvement with their teacher and peers.

Considering the next of the three rings in the theory, (e.g., Renzulli 1978, 1988, 2002, 2016), creativity also plays a prominent role in understanding the development of gifted children in particular, but also, by extension, the optimization of the potentialities of any child regardless of his intellectual abilities at the outset. Creativity is a component of intelligence which is not unrelated to academic success and is as fundamental as it is difficult to identify (Gajda 2016). After having been under debate, creativity is today consensually defined as a cognitive ability to produce content that is not only original (i.e., this content differs from what we usually encounter), but is also coherent with contextual and situational constraints (Lubart et al. 2015; Lubart et al. 2011). These two criteria must be met in order for the content to be judged as neither banal nor strange, but indeed creative (Lubart et al. 2015). The theory of multivariate creativity (e.g., Besançon et al. 2006), which particularly attracted our attention in the creation of this study, is based on the joint influence of cognitive, conative, and environmental factors on the development and expression of creativity. This theory explains the creative process as being governed by two distinct but complementary modes of thinking: exploratory divergent thinking, which is a true generator of multiple ideas, followed by integrative divergent thinking, which acts more as a catalyst for ideas leading to finalized content (Lubart et al. 2011). The creative component of Renzulli’s three-ring model (Renzulli 1978, 1988, 2002, 2016) has therefore been explored in this work through the multivariate approach to creativity (Sternberg and Lubart 1995).

Therefore, because the importance of creativity in child development within the school environment cannot be overlooked, and more particularly as we direct our attention to the population other than gifted children, this study also takes into account the notion of abstract graphic creativity to examine differences between gifted and non-gifted children at school. In the multivariate approach, creativity is domain-dependent (e.g., Shumakova 2018). However, only measures of abstract graphic creativity were collected in this study: this aspect of creativity was supposed to better fit with the context in which both gifted and non-gifted children were encountered (i.e., during art activities performed at school). Creativity is frequently mentioned by practicing psychologists and, more widely, by adults who work with the gifted children. It is even considered by some authors (e.g., Treffinger 1980; Naglieri and Kaufman 2001; Besançon et al. 2006) as one of the components of giftedness (Lubart et al. 2011), although the nature of the relationship between giftedness and creativity is not widely agreed upon (Guignard et al. 2010, 2016).

The three-ring theory of Joseph Renzulli (e.g., Renzulli 1978, 1988, 2002, 2016) also takes into account very superior intellectual abilities to understand giftedness, but not a threshold of 130 of TIQ. However, the purely psychometric conception of the phenomenon, based on the unique criterion of IQ, is disputed (e.g., Carman 2013; Grégoire 2009; Pereira-Fradin et al. 2010). It goes against both the interactional conception of the phenomenon by Renzulli and also the contemporary multi-dimensional conception of intelligence and giftedness which most theorists support (e.g., Liratni and Pry 2007; Fernández et al. 2017). The TIQ threshold of 130 long used for the detection of giftedness raises questions in particular because of the heterogeneity of IQ which seems to characterize gifted children in particular (Liratni and Pry 2007; Pereira-Fradin et al. 2010; Quartier et al. 2019). Rather than a fixed framework where giftedness is exclusively associated with an IQ greater than or equal to 130, a more open approach, starting from 120 or 125, seems essential to meet the needs of children who do not all reach said score at the time of the test but present all the developmental signs characteristic of giftedness.

### 1.3. The Gifted Pupils

As for any population with atypical development, it seems legitimate to ask whether current schooling conditions allow appropriate care for gifted children. Indeed, good or even very good cognitive abilities, as revealed by standardized tests, do not prevent some of these children from underachieving at school and/or receiving poor grades (Kolb and Jussim 1994). They also do not prevent them from being insufficiently stimulated (Reis and Renzulli 2010). The aforementioned asynchronous development of the gifted child undoubtedly explains in large part why these children are not exempt from the possibility of encountering difficulties and/or underachieving at school. These difficulties can be academic (i.e., poor performance, lack of commitment, or methodological problems) or purely adaptive (i.e., social; Desombre et al. 2008). However, because the degree of well-being of the gifted child seems to be associated with his academic success (Kroesbergen et al. 2016), it is necessary to pay particular attention to how he functions as a pupil.

In France, the Education Code provides for special consideration of gifted children. Thus, French schools are theoretically obliged (i.e., in the text) to promote the full development of their potential via specific arrangements, such as acceleration (Steenbergen-Hu and Moon 2011), implementation of a differentiated pedagogical strategy, tutoring, enrichment through using the enrichment triad model (ETM; Kim 2016; Reis et al. 2021; Reis and Peters 2021; Reis et al. 2021), and/or decompartmentalization (MEN 2019). However, as suggested when defining giftedness, the aim is to consider each pupil in his intraindividual differences, beyond the detection of giftedness, thereby adopting a differentiated educational approach within the framework of a coherent partnership between the school and the family to best support his development and flourishing in the school environment.

Today in France, it is necessary to recall that to our knowledge, there are no reliable quantitative data on the prevalence of gifted children with academic difficulties. However, thanks to the substantive work of the CNAHP alone (National Center for Assistance to Children and Adolescents with High Intellectual Potential), conducted with 611 gifted children, it has been established that among those children who consult a psychologist, 76.6% of them are brought there because of problems related to their schooling (Tordjman and Kermarrec 2019). These problems can be behavioral, psycho-affective, related to an associated learning disorder, or (less frequently) related to academic failure (7.5%). Since the CNAHP is linked to a hospital structure, it is important to remember that the malaise at school of these children is not necessarily attributable to their giftedness and may have other causes. However, these results echo the fact that these schoolchildren and their parents often report the feeling of being misunderstood or poorly perceived at school (e.g., Whitmore 1988; Gerber et al. 2018). It also underscores the need to better understand the way they function and their experiences in the school environment, in order to prevent some of these children from feeling like they do not belong in school, despite their great cognitive abilities. According to the aforementioned figures and studies, it seems likely that the care of gifted children in school settings is still far from optimal.

Ultimately, our approach aims to reconcile Renzulli’s theory with the theories of commitment (i.e., SDT and the theory of self-systemic processes) and multivariate creativity (see Figure 1), using tools developed from these empirical theories to consider the functioning of the gifted schoolchild. The notion of commitment has given rise to the use of two scales to measure Feelings about School (French FAS; Sanchez et al. 2022) and Autodetermination At School (AAS; Sanchez and Blanc 2019). The notion of creativity has been explored through tests of convergent and divergent abstract graphic creativity from the EPoC (Lubart et al. 2011). By considering several facets of child development in a school environment and in a learning situation, this study therefore aims to validate the association of the various aforementioned measurement tools in order to evaluate the commitment, feelings, and abstract graphic creativity at school of gifted children compared to non-gifted. We therefore hypothesize a link between the different measures collected from all the children considered, a prerequisite necessary for comparing the various aforementioned theories (i.e., theory of intelligence, engagement, and multivariate approach to creativity). Overall, we expect a differential between the scores of gifted children and those of children-at-large, in favor of gifted children. In detail, we expect that this differential will be salient for all the sub-dimensions that refer to their cognitive development (creativity, academic commitment, feelings of competence). In accordance with the literature (Coleman and Cross 2005; Cross and Cross 2015; Papadopoulos 2020), on the other hand, we expect an absence of differential scores of gifted children compared to children-at-large for all the sub-dimensions referring to their socio-affective development (i.e., links with peers and with teachers). We thus think we can demonstrate that the developmental asynchrony encountered in the gifted child (Papadopoulos 2020) is also perceptible in the functioning of the gifted schoolchild compared to his non-gifted peers.

## 2. Materials and Methods

### 2.1. Participants

In partnership with the rectorate and the Montpellier Academy district, 14 schools were mobilized for this study. The participants (N = 328; see Table 1) were all children from one of the 52 classes who volunteered to participate in this project during the 2019–2020 school year. Among the 328 children who participated in this study, the youngest, enrolled in grades 1 and 2, were from five to seven years old (*M* = 6.33; *SD* = 0.77), while the oldest, enrolled in grades 3, 4, and 5, were from 8 to 11 years old (*M* = 8.79; *SD* = 0.94). They therefore all received a typical public school education in heterogeneous classes, without the giftedness of the individuals concerned necessarily being recognized nor being the object of special consideration. The parents filled out a parental consent form, sent by the teaching teams, informing them that tests and measurements would be carried out on their children if they gave us their consent. The agreement of the head of school, the Academy, and the rectorate was obtained, in addition to that of teachers, parents, and schoolchildren. Thus, this research was approved beforehand by all strata of the school hierarchy. To guarantee an ethical collection, the information was collected anonymously and then kept confidentially. For the recruitment of children-at-large, we included in this study only children for whom the teachers did not report any neurodevelopmental disorder or learning disorder.

Participant distribution is detailed in two stages because we first considered the sample in its entirety for the first part of this study, which was aimed at exploring the links between the different measurements carried out. For the second part of the study, aimed at comparing the functioning of gifted and non-gifted schoolchildren on the different dimensions evaluated, we chose to create a smaller sample (see Table 2) from the initial sample, in order to conduct the analyses on two groups of identical size.

A pre-detection phase was carried out to identify gifted children so that it was not necessary to test all 328 children in our sample. This pre-detection step consisted of asking teachers to indicate within their list of children those who exhibited signs of giftedness, after having given them the pre-detection forms for giftedness provided by the French Ministry of National Education. This pre-detection was also carried out by the experimenters, with sessions of observing the children in their classrooms (during art activities). Also, while the parents were informed about the realization of an experiment related to giftedness in their child’s class, some directly contacted the researcher in charge of the study to signal possible giftedness in their child. A total of 96 children out of 328 were thus tested after this pre-detection phase. The following were considered gifted: (i) those children for whom we had complete reports showing high intellectual potential, with an IQ greater than or equal to 125; (ii) children who obtained a Short IQ greater than or equal to 125 (Grégoire 2009); and (iii) children who obtained a short IQ difficult to interpret (due to a heterogeneity of the scores on the different subtests) but nevertheless greater than 122, with scores much higher for at least two subtests out of the four subtests administered.

For the second comparative part of the study, we kept the sample of 45 gifted children as is. In order to create a balanced sample with the non-gifted children, we selected 45 non-gifted children from the entire sample. Each gifted child was matched with a non-gifted child according to the following criteria: gender, grade, and age. Sometimes only one non-gifted child was perfectly matched, sometimes more than two children were matched. In the latter case, we randomly selected one of them, so as to have a strict one-to-one matching of the children (see Table 2).

### 2.2. Material

Short IQ: Short form of the WISC-IV. The Short IQ is an abbreviated form of the WISC-IV (Wechsler 2005) developed by Grégoire (2009), which makes it possible to achieve a relatively reliable approximation of a child’s TIQ by having him take only four subtests of the WISC-IV. Selected after a comparison of their correlation metric properties with IQ, the four subtests concerned are Similarities (i.e., in order to estimate the Verbal Comprehension Index), Matrix (i.e., in order to estimate the Perceptual Reasoning Index), Sequence-Letters-Numbers (i.e., in order to estimate the Working Memory Index), and Symbols (i.e., in order to estimate the Processing Speed Index). Once standardized, the distribution of the Short IQ follows a Gaussian curve with values very similar to those of the TIQ, of mean 100.02 and standard deviation 14.98. The Short QI and QIT correlate to 0.92 (*p* < 0.001).

### 2.3. Tools for Measuring Engagement, Feelings, and Graphic Creativity

Two psychometric scales were used (see Table 3): the AAS (Sanchez and Blanc 2019) and the French FAS (Sanchez et al. 2022), which corresponds to the enriched translation of the Feelings about School (FAS; Valeski and Stipek 2001). The AAS is a heteroevaluative scale for measuring teacher engagement in the school environment. The French FAS is a self-evaluative scale which allows for the self-evaluative measurement of the child’s Feelings about School.

*AAS.* The self-evaluative psychometric scale of the AAS (Sanchez and Blanc 2019; see Appendix A) offers the possibility of measuring the child’s commitment to school through the viewpoint of his teacher. It is a multidimensional scale comprising three factors and ten items. It makes it possible to collect an overall score of engagement in the school environment of the child as well as three different sub-scores relating to his performance and intrinsic motivation (F1; α = 0.95) and the social link perceived by the schoolchild’s teacher with his peers (F2; α = 0.90) and the teacher himself (F3; α = 0.83). For example, for the student–teacher bond dimension, one of the items that allowed the teacher to situate the children’s attitude between 0 for “very poorly adjusted” and 10 for “very well adjusted” was the following one: “How would you rate his attitude towards you?”

*SSE* or *French FAS.* The self-evaluative and multidimensional scale of the French FAS (Sanchez et al. 2022; see Appendix B) is an adapted and enriched French-language version of the psychometric scale of the FAS by Valeski and Stipek (2001). With five factors divided into fifteen items, it makes it possible to obtain an overall score for measuring the school experience of the elementary school child. Its five sub-dimensions make it possible to distinctly measure the child’s feelings of competence in art (PCA), literacy (PCL), and mathematics (PCM), as well as the way he feels about his relationship with his teacher (FRT) and his general attitude towards school (GAS). The French FAS has a good internal consistency for each of its dimensions, with values ranging from 0.67 to 0.84 (Brunner and Süβ 2005). During the French validation of the scale version, its internal consistency was evaluated by calculating its composite reliability (Sanchez et al. 2022; Tavakol and Dennick 2011). For example, in one item related to the dimension PCA (i.e., the child’s feelings of competence in art), the experimenter told him, “You can use these sticks to show me how good you are at drawing or painting/art.” Then, the child had to place himself on a Likert-type visual stick scale, between the first stick corresponding to “not good at all” and the fifth stick corresponding to “very good”.

*Abstract graphic creativity through two EPoC subtests.* Based on the approach of multivariate creativity, the EPoC (Lubart et al. 2011), the tool for Evaluating the Creative Potential of the child, was designed to be used from age 5 to 12 years old. The EPoC consists of a battery of verbal and graphic subtests, which evaluate in turn the development of divergent-exploratory thinking and that of convergent-integrative thinking through resorting to stimuli which are sometimes concrete and sometimes abstract. For this multimodal study carried out in schools, it was not materially possible to have all of the children take all of the EPoC subtests. In accordance with the context in which we encountered both gifted and non-gifted children in this study (i.e., during art activities at school), only their divergent-exploratory abstract graphic thinking (EPoC-DG) was collected using a first subtest and their convergent-integrative abstract graphic thinking (EPoC-IG) using a second subtest.

### 2.4. Procedure

*Step 1: Sampling.* The non-gifted children who participated in the experiment were randomly selected from lists of the different classes considered. We checked with the teachers that the children thus chosen at random did not have a pre-existing recognized neurodevelopmental disorder or learning disorder. To distinguish gifted children from children-at-large, we relied on the psychological assessments provided by parents when they had previously had their children take them. For children whose assessment was not available or for whom we had to confirm possible giftedness (evoked by the teacher or the parent or suspected by the experimenters), we had them take the short version of the WISC-IV (short IQ; Grégoire 2009). These tests were carried out individually and by the same experimenter, quietly, during school time and at a time chosen by the teacher.

**Figure 2:**
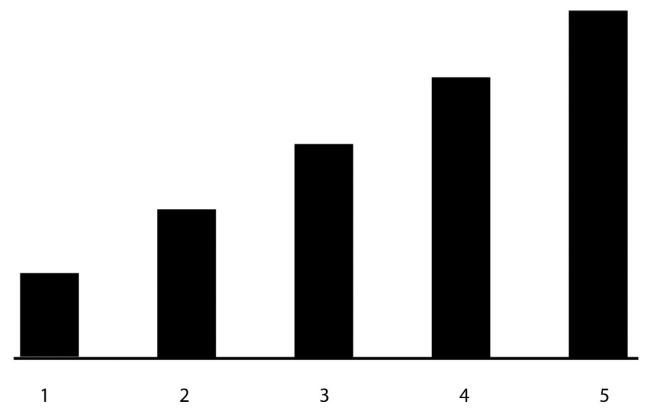
Visualization of the stick scale used for the SSE/French FAS. Figure from Sanchez et al. (2022).

*Step 2: The French FAS.* The self-assessment scale of the French FAS was used at the beginning of the school year with children from 6 to 11 years old. The children, both gifted and non-gifted, responded to the 15 items of the enriched French version of the FAS (Sanchez et al. 2022; Valeski and Stipek 2001). The children were received individually by the experimenter, in a separate room, away from their class. After explaining to the child that all his answers would remain confidential and anonymous, we introduced the French FAS questionnaire by presenting the idea of the visual scale in Likert-type sticks (see Figure 2). The child could then answer the fifteen items of the French FAS. The activity took about ten minutes per child.

*Step 3: The AAS.* At the end of each day of taking the French FAS, the teachers of the children who had been seen for the French FAS were asked to complete the heteroevaluative scale of the AAS. This time, the teachers gave their own point of view on the engagement in the school environment of the schoolchildren considered by filling in the AAS scale for each of them (Sanchez and Blanc 2019). They filled in the scale online, on a secure platform of the laboratory. Teachers had to position a cursor on a continuous line (Allen and Seaman 2007) between 0 and 10 depending on the grade they decided to assign to the schoolchild for each item. Completing the questionnaire took the teacher less than five minutes per schoolchild considered.

*Step 4: The EPoC.* In a third step, we evaluated the same children on two of the abstract graphic tests of the EPoC: the divergent-exploratory graphic thinking test and the convergent-integrative graphic thinking test (Lubart et al. 2011). This time, they were evaluated on their individual graphic productions, in a room separate from their class. They received the instructions of the experimenter in small groups of three to six schoolchildren already included in the experiment. Between the set-up time and the taking of the two tests, each session lasted about 35 min.

## 3. Results

The collected data were analyzed in two stages, and the analyses were mainly carried out using the Jamovi software. All analyses of the study were conducted with a significant threshold of *α* ≤ 0.05, two-tailed.

First, the possible existence of a link between the scores of the AAS, the French FAS, and the two EPoC subtests was examined for the whole sample. The analysis of the correlations carried out for this purpose shows that the different measurement scales used are significantly related to each other, with the exception of the French FAS and the EPoC-DG (*p* = 0.616). Although moderate in intensity, the link is significant between the different dimensions, since Pearson’s *r* oscillates between −0.12 and 0.21. In detail, the French FAS is indeed significantly related to the EPoC-IG (*r* = 0.12; *p* = 0.033; *d* = 0.24) and the AAS (*r* = 0.20; *p* < 0.001; *d* = 0.41). The EPoC-IG is significantly linked to the French FAS, to the EPoC-DG (*r* = 0.20; *p* < 0.001; *d* = 0.42), and to the AAS (*r* = 0.21; *p* < 0.001; *d* = 0.43). The EPoC-DG is significantly linked to the EPoC-IG (*r* = −0.12; *p* = 0.033; *d* = 0.24) and to the AAS (*r* = 0.12; *p* = 0.037; *d* = 0.24). The AAS is significantly linked to the French FAS, the EPoC-IG, and the EPoC-DG.

Second, we systematically compared the scores of the 45 gifted schoolchildren to those of the 45 non-gifted schoolchildren selected in a second step on each of the facets considered (i.e., on the AAS, the French FAS, the EPoC-IG, and the EPoC-DG). As a reminder, for all the ANOVAs carried out as part of the differential analyses conducted, we used the reduced sample, which included the same number of gifted children (n^1^ = 45) as non-gifted (n^2^ = 45; see Table 2). For each ANOVA calculated on the scores obtained from the AAS, the French FAS, and the two EPoC subtests, considered in isolation, the type of schoolchildren (gifted vs. non-gifted), gender (boys vs. girls), and school level (Grades 1 and 2 vs. grades 3, 4, and 5) were considered as intersubject variables. Given that significant differences are expected in this field of research between gifted and non-gifted children, in view of the developmental characteristics of the gifted, it was of importance to explain 10% of the variance. Therefore, it was interesting to identify a η^2^ ≥ 0.10, which corresponds to an *f* of 0.33. Using G*Power software, a sensitivity analysis had been conducted to determine the power of the analyses of variance as a function of the sample size. In the G*Power software, the parameters were: number of groups = 2, α err. prob = 0.05, effect size F = 0.33. The results of this sensitivity analysis indicated that with 90 subjects, there is a statistical power of 87%.

The analyses conducted were mainly aimed at highlighting the effect of the type of children (gifted vs. non gifted). Gender and school level should only be considered as control variables that were taken into account for exploratory purposes. Indeed, the sample size did not allow for a sufficient statistical power to consider these variables as well.

### 3.1. AAS Scores

To observe the effect of the type of schoolchildren (gifted vs. non-gifted) on AAS, we carried out several three-factor ANOVAs, with the AAS score as a dependent variable and then its sub-scores by dimension. On the total score of the AAS, the Levene test reveals homogeneous variances (*p* = 0.297). We observe a main effect of the type of schoolchildren (*F*(1, 82) = 10.121, *p* = 0.002, η^2^ = 0.100), the average of gifted children (*M* = 85.6, *SD* = 7.23) being significantly higher than that of non-gifted children (*M* = 79.5, *SD* = 12.4; see Table 4). Moreover, we do not observe any effect of gender (*p* = 0.744) nor of grade level (*p* = 0.148).

In detail, the main effect of the type of schoolchildren is found for the AAS-PIM sub-score (*F*(1, 82) = 12.995, *p* < 0.001, η^2^ = 0.132), with significantly higher average scores for gifted schoolchildren (see Table 4). This effect of the type of schoolchild is still present for the AAS-ST sub-score (*F*(1, 82) = 5.350, *p* = 0.023, η^2^ = 0.056) but not for the AAS-SP (*F*(1, 82) = 0.01, *p* = 0.753). We also notice a simple effect of grade level on the AAS-ST (*F*(1, 82) = 4.067, *p* = 0.047, η^2^ = 0.043), with an increased commitment of the schoolchild with respect to the teacher for the older ones (*M* = 16.7, *SD* = 3.09) compared to the youngest (*M* = 15.1, *SD* = 3.88).

### 3.2. French FAS Scores

Considering the overall score of the French FAS (see Table 5), with the Levene test revealing homogeneous variances (*p* = 0.089), we observe a main effect of the type of schoolchildren (*F*(1, 82) = 11.089, *p* = 0.001, η^2^ = 0.112), the score of gifted children (*M* = 60.73, *SD* = 6.84) being significantly higher than that of non-gifted children (*M* = 54, *SD* = 11.29). There is also an interaction effect on the French FAS score between giftedness and grade level. The post hoc tests (with Bonferroni correction) reveal that the simple effect of giftedness is larger in younger children (*t* = −3.75, *p* = 0.002) than in older ones (*t* = −2.99, *p* = 0.022). The main effect of giftedness is found in the sub-scores of the scale relating to the perceptions that children have of their skills: for the PCA (*F*(1, 82) = 5.615, *p* = 0.020, η^2^ = 0.058), with a large effect size for the PCL (*F*(1, 82) = 21.119, *p* < 0.001, η^2^ = 0.189), and for the PCM (*F*(1, 82) = 8.308, *p* = 0.005, η^2^ = 0.079). On the other hand, there is no effect of giftedness on the FRT sub-scores (*F*(1, 82) = 1.11, *p* = 0.295) and GAS (*F*(1, 82) = 0.683, *p* = 0.411). Concerning the PCA sub-score, we also note a simple effect of gender (*F*(1, 82) = 6.037, *p* = 0.016, η^2^ = 0.062), with girls (*M* = 11.38, *SD* = 2.99) having a better perception of their art skills than boys (*M* = 9.98, *SD* = 2.90). Regarding the PCM sub-score, we also note a simple effect of gender (*F*(1, 82) = 4.270, *p* = 0.042, η^2^ = 0.072) and grade level (*F*(4, 70) = 2.771, *p* = 0.034, η^2^ = 0.117). The youngest children (*M* = 12.87, *SD* = 2.90) and boys (*M* = 12.8, *SD* = 2) have a better perception of their math skills than older children (*M* = 11.5, *SD* = 1.97) and girls (*M* = 11.62, *SD* = 2.95).

### 3.3. Comparison of the Scores on the Two EPoC Subtests: The EPoC-IG and the EPoC-DG

On the score obtained from the EPoC-DG, there is a main effect of the type of schoolchildren (*F*(1, 80) = 4.367, *p* = 0.040, η^2^ = 0.048), the gifted children (*M* = 4.71, *SD* = 1.31) obtaining significantly higher scores than those of non-gifted children (*M* = 4.12, *SD* = 1.43). The main effect of the type of schoolchildren also emerges considering the EPoC-IG (*F*(1, 80) = 11.530, *p* = 0.001, η^2^ = 0.101), the score of gifted children (*M* = 4.44, *SD* = 1.59) being significantly higher than that of non-gifted children (*M* = 3.37, *SD* = 1.68). A simple effect of gender (*F*(1, 80) = 5.479, *p* = 0.022, η^2^ = 0.048) and grade level (*F*(1, 80) = 12.270, *p* < 0.001, η^2^ = 0.108) also appears on the EpoC-IG. Younger schoolchildren (*M* = 3.35, *SD* = 1.77) and boys (*M* = 3.52, *SD* = 1.80) obtain significantly lower scores than those of older schoolchildren (*M* = 4.55, *SD* = 1.58) and girls (*M* = 4.32, *SD* = 1.58).

## 4. Discussion

This study had two major objectives. First, as a starting prerequisite, it was necessary to validate our bias towards a theoretical framework associating Renzulli’s three-ring theory (e.g., Renzulli 1978, 2002, 2016), engagement theories (Connell and Wellborn 1991; Ryan and Deci 2017), and the multivariate approach to creativity. Second, the aim of this study was to shed further light on the differences in functioning of gifted and non-gifted schoolchildren in terms of commitment, feelings about school, and abstract graphic creativity. Our purpose was to contribute to the literature relating to the particular functioning of gifted schoolchildren, making it possible to better identify these children in order to better accompany them in their schooling.

### 4.1. Relevance and Implications of the Three-Ring Theory in Understanding Schoolchildren’s Development

The results of this study therefore reaffirm the relevance of studying different facets of school-based development in children. On the one hand, the significant results obtained confirm the presence of links between the various measurements carried out; however, caution is necessary in view of the moderate strength of the observed link to the whole sample. The child’s commitment as perceived by his teacher (AAS), his feelings about school (French FAS), and his convergent-integrative and abstract creative thinking appear to be effectively linked. These results are consistent with the three-ring theory of Renzulli (e.g., Renzulli 1978, 2002, 2016), which conceives that the harmonious development of the learner is ternary, requiring intellectual skills, commitment, and creativity. These results also show that it is possible to integrate the theories of engagement (i.e., SDT and autosystem process theory) and the multivariate creativity approach into Renzulli’s three-ring conception of intelligence. On the other hand, the measurement of abstract divergent-exploratory creative thinking and that of feelings about the child’s school are not correlated. Although the measure of divergent creativity is only graphic here and does not make it possible to conclude that there is no link between the child’s experience of school and the development of his divergent thinking in a more global way, the fact remains that this absence of correlation raises questions. Indeed, divergent thinking is the expression of the child’s ability to generate a maximum of ideas from a single starting point. Consequently, it requires a particularly fluid and flexible functioning of thought that is underemphasized in the French educational model (Lubart and Mouchiroud 2003), which is still very vertical. Therefore, could we explain the fact that the non- or underdeveloping of this skill is unrelated to feelings about the child’s school by the importance that is given to the development of divergent thinking in the school environment? The question remains open, but it is indeed possible that the underdevelopment of this divergent thinking does not impact the Feelings about School, because the child does not perceive the need to develop this ability based on the expectations of the school. It would be counterintuitive indeed to think that this result can be explained by the idea that the development of divergent thinking does not have a role to play in child development in the short, medium, and long term. Divergent-exploratory thinking seems fundamental, in particular to well-being and fulfillment in a professional environment (e.g., Sternberg and Lubart 1995, 1996), with later repercussions on individual growth. Indeed, the generation of multiple ideas (via divergent thinking) leads to the elaboration and culmination of motor ideas, through the mediation of flexibility which is then transformed into a convergent or synthetic mode of thinking.

### 4.2. The Teacher’s Perception of the Gifted Pupil’s Commitment

Regarding the comparison of AAS scores between gifted and non-gifted children, this study made it possible to highlight, from the teacher’s point of view, a greater sense of self-determination at school for gifted schoolchildren. The superiority of the gifted child’s engagement in the school environment thus reaffirms the relevance of Renzulli’s three-ring model of intellect, intelligence, and giftedness (e.g., Renzulli 1978, 2002, 2016), while at the same time contradicting the myth of systematically problematic engagement at school found in certain media and social representations of gifted schoolchildren (e.g., Sanchez et al. 2022). Indeed, teachers evaluate the academic performance and the intrinsic motivation of gifted children more favorably than their non-gifted peers; they therefore seem to perceive the academic and communicative over-efficiency of the gifted child. Teachers evaluate even more favorably the bond they maintain with their gifted schoolchildren and their oldest schoolchildren, as compared with their non-gifted schoolchildren and their youngest ones (i.e., from grades 1 and 2). Due to the small sample size, the grade-level effect observed has to be cautiously explained: It seems that the engagement of the youngest children is very dependent on the quality of the emotional bond maintained with the teacher (e.g., Furrer and Skinner 2003). The more positive teacher–schoolchild relationship for the gifted schoolchild is surprising in view of literature which points to relationships that may be altered between this population of schoolchildren and their teachers (e.g., Kolb and Jussim 1994), explained in particular by the erroneous social representations and differentiating stereotypes that they nourish with regard to the gifted as a stigmatized social group in general (Jussim and Harber 2005; Lorenz 2021; Lopez and Sotillo 2009; Sanchez et al. 2022). It may be that the result revealing a favorable teacher–gifted schoolchild relationship is attributable to the fact that we have an underrepresentation of under-achieving gifted children in our sample, the pre-detection of the gifted having been done by the teachers, who identified them primarily on the basis of their academic success. However, in this study, the teachers were mostly unaware that the schoolchildren considered to be gifted actually were. It may therefore be that without the “labeling” effect, without knowing if a child is gifted, they have a more positive and fair vision of this child which is devoid of stereotypes. It may also be that the teachers’ sense of personal self-efficacy, which can have an influence on their perception of schoolchildren and the stereotypes likely to be associated with them (Knigge et al. 2016), is not impacted when there is no label that would highlight their possible lack of training and ability to take care of the gifted child. Without a label, the gifted child is certainly overall an above-average and more successful schoolchild who may benefit from a better teacher–schoolchild bond than his peers, like any “good” schoolchild (e.g., Kolb and Jussim 1994). However, it does not result in an idealized vision of better commitment for the child in all areas, since there is no effect of giftedness on the dimension evaluating the commitment of the child in his relationship with peers. This is a result that is consistent not only with increased cognitive development in the gifted child allowing him to perform better and to exhibit a greater intrinsic motivation with regard to learning, but also with the observation that gifted children present socio-emotional and affective development that is not decreased, but well-assimilated to the norm by comparison with non-gifted individuals of the same chronological age (e.g., Papadopoulos 2020; Schwean et al. 2006; Shechtman and Silektor 2012), or even slightly increased (Abdulla Alabbasi et al. 2021; Ogurlu 2021). The contribution of this study concerning this aspect should not make the idea less salient that asking teachers to rate children’s engagement may lead to incorrect conclusions, since schoolchildren may have different emotional and behavioral engagement levels, and there are multiple reasons why a schoolchild may not demonstrate engaged behavior.

### 4.3. Focus on Gifted Children’s Feelings about School

When they self-evaluate their Feelings about School (Sanchez et al. 2022; Valeski and Stipek 2001), gifted children overall present significantly better scores than those of their peers. A detailed observation of the comparison of scores between gifted and non-gifted children leads less to an idealized vision of the gifted child, who apparently does not feel less bad, but downright better than his peers at school, whom he encourages to adopt a nuanced vision on what is going better and what is going “normally” at school for these children. Indeed, the main effect of giftedness stands out for the three dimensions that affect the child’s perception of his academic skills in mathematics, literacy, and art. The gifted child therefore seems to consider himself better than his peers in terms of purely academic or academic performance, seemingly with good reason given his greater cognitive abilities. This result is supported in the literature by other studies that show that the gifted child’s academic self-esteem is better than that of his peers at school (e.g., Hoge and Renzulli 1993), although it is known that when gifted children have academic results that are not up to their cognitive faculties, they tend to have a poorer image of themselves and lower self-esteem (Dowdall and Colangelo 1982; Kolb and Jussim 1994). On the other hand, on the two dimensions of the scale that indirectly affect the socio-affective skills of the child, through the evaluation of his connection with his teacher and his general perception of the school, the gifted children’s scores are normalized. It therefore seems that here again, the distinction between the increased cognitive development and the normalized socio-affective development of the gifted child is confirmed (Papadopoulos 2020; Terrassier 2009). Gifted children with academic difficulties also report difficult relationships with others (Courtinat and de Léonardis 2006). It is interesting, however, that teachers evaluate their relationships with gifted children as better than the ones they maintain with children-at-large, while gifted children do not evaluate their relationship with their teachers more favorably than others. There is therefore a perceptual divide here, which could possibly be explained by higher expectations of gifted children with regard to the emotional bond they form with their teachers and with regard to school in general, where they are not necessarily stimulated to the height of their abilities (e.g., Kolb and Jussim 1994; Siegle and McCoach 2018). This could explain, on the other hand, why they do not believe that their teachers feel that they have a more qualitative link with them than with their peers. Other effects of grade level and gender appear in detail but have to be considered with caution due to the small sample size. Girls seem to have a better perception of their art skills than boys. Without much surprise (e.g., Dutrévis and Toczek 2007), we note an opposite effect concerning the perception that the child has of his skills in mathematics, with boys who evaluate themselves more positively than girls. The two aforementioned observations can be linked to the existence of gender stereotypes associated with school disciplines, as well as to the impact of these stereotypes on the perception that children and teachers have as to the supposed superior skills of girls in art and boys in mathematics (Dutrévis and Toczek 2007). The perception of mathematics skills is further modulated by the school level, since the youngest children consider themselves more competent in mathematics than their older peers, a result that reaffirms the idea that the youngest children may have an exaggerated and still imprecise perception of their real skills in mathematics, since this exaggerated perception was a priori attributable to a set of factors such as the increased encouragement of teachers of small classes, a lesser comparison with peers, a differentiated way of managing the class, and academic tasks in front of an audience of “small” (e.g., Newman 1984). Finally, it emerged from this study that the youngest gifted children seem to stand out more from children-at-large than their older peers. This differential in French FAS scores, between the youngest and the oldest, had also been encountered in children-at-large at the time of validation of the tool (Sanchez et al. 2022). Despite the need to replicate this result on a larger sample, it is interesting that this already observed phenomenon of decreasing academic well-being from the age of 10 (Casas and González-Carrasco 2019) can be found in an exacerbated way in children-at-large.

### 4.4. Nature of the Abstract Graphic Creativity of Gifted Children Compared to Their Non-Gifted Peers

Despite the moderate size of our sample regarding the number of gifted children (n = 45), this study also tends to provide evidence that gifted children perform better than their non-gifted peers when it comes to abstract graphic creativity, whether it is graphic integrative-convergent thinking or graphic exploratory-divergent thinking. These results complement those obtained during the creation of the EPoC, during which gifted children obtained better results in terms of exploratory-divergent thinking but not in terms of integrative-convergent thinking for abstract stimuli (Lubart et al. 2011). Moreover, they corroborate the observation already made of the superiority of the gifted in terms of convergent thinking (Besançon et al. 2011; Guignard and Lubart 2007) as well as divergent (Fernández et al. 2017). These are results that in fact reaffirm the importance of considering creative ability as an integral part of the particularly efficient cognitive development of gifted individuals. These results further show that creativity can be an interesting gateway to establishing the developmental balance of gifted children, in the school environment as well as outside. This can indeed make it possible to satisfy their appetite for novelty as well as maintain their motivation, while at the same time cultivating their talents (i.e., by giving them the possibility of expressing them, or even revealing them, in several directions). Our results also tend to show that girls score better than boys in terms of abstract graphic integrative creativity, in contrast with the absence of a gender effect observed at the time of the creation of the EPoC (Lubart et al. 2011). It would therefore be interesting to see if subsequent studies find this superiority of girls in terms of convergent-integrative thinking. Moreover, we observe, unsurprisingly in view of the results obtained by Lubart et al. (2011) during the creation of the test, that the youngest children in the sample have worse results than their older peers in terms of convergent-integrative abstract graphic creativity (Lubart et al. 2011).

### 4.5. Major Contributions and Perspectives

Overall, this study tends to show that gifted children are more engaged in school, feel more successful in everything related to their intellectual development, and have better abstract graphic creativity compared to their non-gifted peers. It also appears that gifted children have the feeling that their socio-emotional development does not follow the same pattern, as evidenced by their perception of their social bond with their teacher or by the teacher’s perception of their commitment in the relationship with their peers. The perception that teachers have of their own socio-emotional commitment is however more nuanced, since they consider that their connection with gifted schoolchildren is better than with their non-gifted peers and believe that their social commitment with their non-gifted peers is within the norm. The perceptual gap revealed in the teacher–child relationship is an interesting way to help teachers and schoolchildren improve their mutual communication in order to achieve a more reassuring relationship for both of them. Moreover, the result relating to the progressive decrease in the gifted child’s feelings about school according to age group, compared to their non-gifted peers, also seems very important to understand the functioning of the gifted child. However, this finding needs to be replicated in future studies to ensure that a larger decline in school well-being can be confirmed for gifted children. Then, it will be essential to find ways to address this issue to support their healthy development.

## 5. Conclusions

This study reaffirms the necessity of considering a child’s commitment, feelings, and creativity for his development and fulfillment in the school environment. It also tends to demonstrate that the development of gifted children in the school environment is significantly more favorable compared to that of children-at-large with regard to their academic commitment, their positive attitudes towards school, and their abstract graphic creativity in terms of convergent and divergent thinking. In any case, the results obtained point away from a pathologizing comprehension of the functioning of gifted children in a school setting. In addition, the absence of a differential between gifted and non-gifted children considering the socio-affective sphere reaffirms normalized development in these same children and, consequently, a discrepancy between their cognitive and socio-affective development. Thus, even if they are generally doing quite well at school, this does not free us from a duty to be vigilant given this developmental asynchrony, in order to promote more equitable and harmonious support aimed at avoiding difficulties for them at school likely to impair their development.

## 6. Limits and Perspectives

The fact that some of the children in this study were considered gifted by only doing a short IQ using the WISC IV can be understood as a limit to the generalization of the results of this study. It is possible that the gifted schoolchildren in the sample, mostly pre-detected by their teachers or identified by screenings, are also good schoolchildren and that we have missed out on gifted schoolchildren in difficulty or failing school, whom it would have been good to be sure to have included for a more representative panel of gifted schoolchildren in their diversity. However, this limit should be put into perspective since, on the one hand, the IQ tests performed in their entirety are also sensitive to multifactorial intraindividual variations and, on the other hand, because the significance of the results tends to validate the recruitment method used, since it made it possible to reveal interindividual differences between the two distinct populations we studied.

One other limit to the generalization of the results reported in this study also lies in its sampling, since the sample size of gifted children is still too small to overcome the statistical power of the analyses conducted. Regarding the links reported between the various measurements collected in this study, on the basis of the three-ring theory of intelligence (Renzulli 1978, 1988, 2002, 2016), which initially focused on understanding giftedness, it seems plausible to hypothesize that the correlations could be stronger for gifted children. This hypothesis deserves to be tested with a larger sample of gifted children, which was not possible in the present study, for a robust comparison of the differences that might be observed in correlational links between gifted and non-gifted children

Another limitation to this work is due to the fact that the measurement of creative thinking, for purely ecological reasons, was reduced to abstract graphic measurements only. It would be interesting to further examine in the future to what extent this divergent thinking, comprehended in its entirety through complementary tests of the EPoC, with figurative and abstract stimuli, in both verbal and graphic spheres, is still not correlated with feelings about school in relation to integrative creative thinking.

Beyond the perspectives which this study opens up in order to better understand gifted children, we believe that the result relating to a decrease in academic well-being with age, through the measurement of Feelings about School and which was greater in gifted children than their non-gifted peers, invites us to carry out other research in the future. Other facets of the social and emotional development of gifted children should be further examined to confirm this phenomenon, explain it, and if necessary, to try to overcome it in order to ensure better development of the gifted child in elementary school.

## Figures and Tables

**Table 1:** Descriptive statistics of the overall sample used in the study.

Children	Gifted (n^1^)	Non-Gifted (n^2^)	Total (N)
Total	45	283	328
Male	23	143	166
Female	22	140	162
Grade (Age)			
Grades 1/2 (age 5 to 7)	23	133	156
Grades 3/4/5 (age 8 to 11)	22	150	172

n^1^: number of Gifted Children; n^2^: number of Non-Gifted Children.

**Table 2:** Descriptive statistics for the smaller homogeneous sample used in the study.

Children	Gifted (n^1^)	Non-Gifted (n^2^)	Total (N)
Total	45	45	90
Male	23	22	45
Female	22	23	45
Grade (Age)			
Grades 1/2 (age 5 to 7)	23	23	46
Grades 3/4/5 (age 8 to 11)	22	22	44

n^1^: number of Gifted Children; n^2^: number of Non-Gifted Children.

**Table 3:** Description of the factorial structures of the AAS and French FAS (i.e., SSE) scales.

3 Factors for the AAS	5 Factors for the SSE *
F1:PIM (Performance and Intrinsic Motivation)	F1:PCA (Perceived competence in art)
F2:SP (Schoolchild–peers)	F2:PCL (Perceived competence in literacy)
F3:ST (Schoolchild–teacher)	F3:PCM (Perceived competence in math)
	F4:FRT (Feelings about relationship with teacher)
	F5:GAS (General attitudes toward school)

* French-enriched translation of the FAS scale of (Valeski and Stipek 2001; Feelings about School; Sanchez et al. 2022).

**Table 4:** Comparison of the scores of gifted and non-gifted on the AAS, with means (M), standard deviations (SD), and *p*-value.

Factors of the AAS	Gifted *M* (*SD*)	Non-Gifted *M* (*SD*)	*p*
AAS (Global score)	85.6 (7.23)	79.5 (12.4)	0.002
AAS-PIM (Performance and Intrinsic Motivation)	50 (8.49)	36.7 (8.29)	<0.001
AAS-ST (Schoolchild–teacher)	19 (2.23)	14.125 (4.12)	0.023
AAS-SP (Schoolchild–peers)	14.3 (4.55)	17.14 (4.88)	0.753

**Table 5:** ANOVAs: Comparison of the scores of gifted and non-gifted children on the French FAS (i.e., SSE), with means (M), standard deviations (SD), and *p*-value.

Factors of the SSE	Gifted *M* (*SD*)	Non-Gifted *M* (*SD*)	*p*
SSE (Overall score)	60.73 (6.84)	54 (11.30)	0.001
PCA (Perceived competence in art)	11.4 (2.53)	9.96 (3.31)	0.020
PCL (Perceived competence in literacy)	12.51 (2)	9.78 (3.38)	<0.001
PCM (Perceived competence in math)	12.93(1.85)	11.47(2.97)	0.005
FRT (Feelings about relationship with teacher)	11.31(2.27)	10.69(3.02)	0.295
GAS (General attitude toward school)	12.58(1.92)	12.11(3.16)	0.411

## Data Availability

The data presented in this study are available on request from the corresponding author. The data are not publicly available due to privacy.

## Appendix A

The AAS scale, associated items, and factors.

**The AAS’ Items**	**Associated Factors**
How would you rate his level of achievement in mathematics? [Comment évaluez-vous son niveau de réussite en mathématiques?] (*0 = far below; 10 = far above)*	Performance and Intrinsic Motivation (*F*1)
How would you rate his attitude towards you? [Comment évaluez-vous son attitude à votre égard?] *(0 = very poorly adjusted; 10 = very well adjusted)*	Child–teacher bond (*F*3)
How would you rate his ability to integrate himself into the class group? [Comment évaluez-vous sa capacité d’intégration au groupe classe?] (*0 = very bad; 10 = very good)*	Child–Peer bond (*F*2)
How do you think he feels when you place him in a learning situation? [Que pensez-vous qu’il éprouve lorsque vous le placez en situation d’apprentissage?] (*0 = a lot of displeasure; 10 = a lot of fun)*	Performance and Intrinsic Motivation (*F*1)
How do you evaluate his writing from a graphic point of view? [Comment évaluez-vous son écriture d’un point de vue graphique?] *(0 = very laborious; 10 = very neat)*	Performance and Intrinsic Motivation (*F*1)
Would you say that this student can work independently? [Diriez-vous de cet élève qu’il arrive à travailler en autonomie?] (*0 = never; 10 = all the time tout le temps)*	Performance and Intrinsic Motivation (*F*1)
How would you rate his attitude towards other children in the class? [Comment évaluez-vous son attitude à l’égard des autres élèves de sa classe?] *(0 = very poorly adjusted; 10 = very well adjusted)*	Child–Peer bond (*F*2)
How would you rate his level of success in literature? [Comment évaluez-vous son niveau de réussite en français?] (*0 = far below; 10 = far above)*	Performance and Intrinsic Motivation (*F*1)
Is this student discouraged by schoolwork? [Cet élève se décourage-t-il face au travail?] *(0 = all the time; 10 = never)*	Performance and Intrinsic Motivation (*F*1)
How would you rate your relationship with the student? [Comment évaluez-vous le lien que vous entretenez avec cet élève?] *(0 = very poorly adjusted; 10 = very well adjusted)*	Child–teacher bond (*F*3)

## Appendix B

The French FAS (i.e., SSE) scale, associated items and factors.

**The French FAS’ Items**	**Associated Factors**
You can use these bars to show me how much your teacher cares about you. [Tu peux utiliser ces bâtonnets pour me montrer à quel point ton maître/ta maîtresse s’occupe de toi.] *(1 = Doesn’t care at all; 5 = Care a lot)*	Feelings about teacher (*F*4)
You can use these sticks to show me how good you are at drawing or painting/art. [Tu peux utiliser ces bâtonnets pour me montrer à quel point tu es fort en dessin ou peinture/art.] *(1 = Not at all good; 5 = Very good)*	Perceived competence in art *(F1)*
You can use these bars to show me how much you know about reading. [Tu peux utiliser ces bâtonnets pour me montrer à quel point tu sais lire/comprends ce que tu lis.] *(1 = Don’t know much at all; 5 = Know a lot)*	Perceived competence in literacy (*F*2)
You can use these bars to show me how you feel about going to school. [Tu peux utiliser ces bâtonnets pour me montrer comment tu te sens quand tu vas à l’école.] *(1 = Like going to school a lot; 5 = Don’t like going to school at all)*	General attitudes toward school (*F*5)
You can use these bars to show me how your teacher feels about you. [Tu peux utiliser ces bâtonnets pour me montrer à quel point ton maître/ta maîtresse tient à toi.] *(1 = Doesn’t like you at all; 5 = Likes you a lot)*	Feelings about teacher (*F*4)
You can use these bars to show me how fun the things you do in school are. [Tu peux utiliser ces bâtonnets pour me montrer à quel point ce que tu fais à l’école est amusant.] *(1 = Not fun at all; 5 = Very fun)*	General attitudes toward school (*F*5)
You can use these bars to show me how good you are at numbers/math. [Tu peux utiliser ces bâtonnets pour me montrer à quel point tu es fort avec les nombres/en maths.] *(1 = Not good at all; 5 = Very good)*	Perceived competence in math (*F*3)
You can use these bars to show me how much you know about letters. [Tu peux utiliser ces bâtonnets pour me montrer à quel point tu sais écrire.] *(1 = Don’t know much at all; 5 = Know a lot)*	Perceived competence in literacy (*F*2)
You can use these bars to show me how you feel when you are at school. [Tu peux utiliser ces bâtonnets pour me montrer comment tu te sens quand tu es à l’école.] *(1 = Not good at all; 5 = Very good)*	General attitudes toward school (*F*5)
You can use these sticks to show me how much you know about drawing or painting/art. [Tu peux utiliser ces bâtonnets pour me montrer tout ce que tu sais en dessin ou peinture/art.] *(1 = Don’t know much at all; 5 = Know a lot)*	Perceived competence in art *(F1)*
You can use these bars to show me how much you know about numbers/math. [Tu peux utiliser ces bâtonnets pour me montrer tout ce que tu sais sur les nombres/en maths.] *(1 = Don’t know much at all; 5 = Know a lot)*	Perceived competence in math (*F*3)
You can use these bars to show me how good you are at reading. [Tu peux utiliser ces bâtonnets pour me montrer à quel point tu es fort en lecture/français.] *(1 = Don’t know much at all; 5 = Know a lot)*	Perceived competence in literacy (*F*2)
You can use these bars to show me how you feel about your teacher. [Tu peux utiliser ces bâtonnets pour me montrer à quel point tu tiens à ton maître/ta maîtresse.] *(1 = Don’t like at all; 5 = Like a lot)*	Feelings about teacher (*F*4)
You can use these bars to show me how good you are learning something new in numbers. [Tu peux utiliser ces bâtonnets pour me montrer à quel point tu te sens fort pour apprendre quelque chose de nouveau surles nombres/en maths.] *(1 = Not good at all; 5 = Very good)*	Perceived competence in math (*F*3)
You can use these bars to show me how good you are learning something new in drawing or painting/art. [Tu peux utiliser ces bâtonnets pour me montrer à quel point tu te sens fort pour apprendre quelque chose de nouveau en dessin ou peinture/art.] *(1 = Not good at all; 5 = Very good)*	Perceived competence in art *(F1)*
